# Balancing Microglial Density and Activation in Central Nervous System Development and Disease

**DOI:** 10.3390/cimb47050344

**Published:** 2025-05-09

**Authors:** Shunqi Wang, Liangjing Pan, Chong Sun, Chaolin Ma, Haili Pan

**Affiliations:** 1 The Second Affiliated Hospital, Institute of Biomedical Innovation, Jiangxi Province Key Laboratory of Brain Science and Brian Health, and School of Basic Medical Sciences, Jiangxi Medical College, Nanchang University, Nanchang 330031, China; wsqi@ncu.edu.cn (S.W.); panliangjing2023@163.com (L.P.); sunc@ncu.edu.cn (C.S.); 2 School of Life Sciences, Nanchang University, Nanchang 330031, China; 3 Institute of Pain Medicine, Institute of Nautical Medicine, Nantong University, Nantong 226019, China

**Keywords:** microglia, neurogenesis, synaptic pruning, neurological diseases, therapeutic interventions, lifestyle modifications

## Abstract

Microglia, the resident immune cells of the central nervous system, play multifaceted roles in both health and disease. During development, they regulate neurogenesis and refine neural circuits through synaptic pruning. In adulthood, microglia maintain homeostasis and dynamically respond to pathological insults, where they contribute to responding to neuroinflammatory challenges. This review summarizes microglial contributions to neurodevelopment and also outlines their function across various neurodegenerative diseases, such as Alzheimer’s disease, Parkinson’s disease, Huntington’s disease, and amyotrophic lateral sclerosis, highlighting both protective and detrimental effects. Finally, recent advances in microglial-targeted therapies and lifestyle-based interventions are highlighted, underscoring the translational potential of modulating microglial states. Elucidating the dual roles of microglia in development and disease could guide the design of therapeutic strategies aimed at enhancing neuroprotection while minimizing neurotoxicity.

## 1. Introduction

Microglia, the innate immune cells of the central nervous system (CNS), are essential for maintaining neural homeostasis, modulating neurodevelopment, and responding to neurological disease. During early development, microglia participate in neurogenesis and play an essential role in synaptic pruning, ensuring proper connectivity within the brain. Historically, research has largely focused on the consequences of microglial activation, often portraying these cells as mediators of neuroinflammation and neurodegeneration. However, a more nuanced understanding has emerged, recognizing that microglia exhibit diverse functional states depending on their environment and specific stimuli.

Reduction in microglial density is often in the CNS development or disorders and microglial depletion has been associated with a range of CNS pathologies, highlighting the importance of maintaining an optimal microglial population for CNS health. For instance, microglial density is critical for efficient synaptic pruning during development, and its reduction can lead to impaired cognitive functions and increased vulnerability to neurodegenerative diseases. As our understanding of microglial biology deepens, it becomes clear that an exclusive focus on microglial density reduction would be insufficient to capture the full spectrum of their roles in the CNS. Therefore, this review was expanded to include studies on microglial density increase and activation. Increased microglial density, often observed in neuroinflammatory and neurodegenerative conditions, suggests a compensatory or pathological response to CNS insults. Moreover, the activation states of microglia, ranging from pro-inflammatory to anti-inflammatory phenotypes, play pivotal roles in disease progression and resolution.

In this comprehensive review, we resume the roles of microglia in neurodevelopment, with a particular emphasis on synaptic pruning and neurogenesis. And then summarize the dual roles of microglia in various neurodegenerative diseases, including Alzheimer’s disease (AD), Parkinson’s disease (PD), Huntington’s disease (HD), and amyotrophic lateral sclerosis (ALS), highlighting both protective and detrimental effects. Finally, we critically evaluate therapeutic strategies targeting microglia and lifestyle modifications, further underscoring non-invasive avenues to optimize microglial function. Unraveling microglial heterogeneity and activation dynamics will be pivotal in developing precision therapies for neurodegenerative and neuroinflammatory diseases.

## 2. Microglial Function in Neurodevelopment

Microglia serve as central regulators of neurodevelopmental processes through their dynamic interactions with neural elements. Beyond their canonical immune functions, they orchestrate neurogenesis events in the development or the pathological contexts, and synaptic pruning in neural circuit optimization [1,2,3,4].

### 2.1. Regulation of Neurogenesis

**Figure 1:**
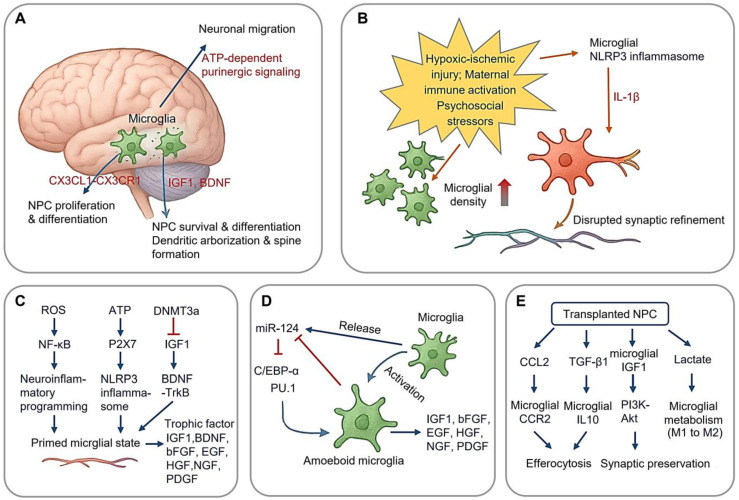
Microglial Regulation on Neurogenesis. (**A**) Microglia regulates neurogenesis in ventricular–subventricular zone (V-SVZ); (**B**) perinatal stress induces neuroinflammatory programming, increase in microglial density, and disrupted synaptic refinement of neuron; (**C**) stress-induced epigenetic changes and neuroinflammation of microglia; (**D**) microglial miR124 and neurogenesis; (**E**) modulation of NPC-microglia crosstalk in NPC transplantation, microglial metabolism from pro-inflammatory glycolysis (M1) to OXPHOS-dominated anti-inflammatory state (M2). (Arrow: activation; red blunt arrow: inhibition; red thick arrow in panel (**B**): increase).

Microglia rapidly proliferate during the perinatal and postnatal stages, fully colonizing the brain by the end of the second postnatal week [5,6]. Disruptions during this critical window can have lasting consequences on brain health [7,8]. Simultaneously, microglia facilitate neurogenesis by regulating the ventricular–subventricular zone niche through secretory signaling (Figure 1A). Via CX3CL1 (Chemokine C-X3-C-Motif Ligand 1)-CX3CR1 (C-X3-C Motif Chemokine Receptor 1) signaling, they modulate neural progenitor cell (NPC) proliferation and differentiation, with genetic ablation experiments demonstrating 30% reductions in newborn neurons in the absence of CX3CR1 [9]. Postnatally, microglial-derived growth factors, including IGF1 (Insulin-like Growth Factors-1), BDNF (brain-derived neurotrophic factor), promote dendritic arborization and spine formation. Additionally, microglia release ATP to activate purinergic signaling pathways (e.g., P2Y receptors), which guide neuronal migration through chemotactic gradients [10].

#### 2.1.1. Perinatal Stress and Neuroinflammatory Programming

Perinatal stress, encompassing physiological or psychological insults occurring from mid-gestation and the early postnatal period, has been implicated in cognitive deficits and psychiatric disorders, potentially via altered microglial function during development [11,12,13,14]. Three perinatal stress paradigms dominate developmental studies (Figure 1B): hypoxic–ischemic injury, maternal immune activation, and psychosocial stressors (e.g., maternal separation). These distinct stressors converge on microglial NLRP3 (NLR family pyrin domain containing three) inflammasome activation, leading to IL-1β (Interleukin-1β) production, which disrupts synaptic refinement [15].

Acute stress has been shown to increase microglial density in the hippocampus of rat pups, an effect that may compound activation induced by perinatal asphyxia [16]. This increase is particularly pronounced in the hippocampal CA1 region 24 h post-stress exposure through CX3CR1-dependent chemotaxis, suggesting a rapid microglial response [16,17]. This rapid mobilization is accompanied by a morphological shift from a surveillance state to an amoeboid phagocytic state [18]. Notably, the “double-hit” hypothesis posits those perinatal insults prime microglia for exaggerated responses to subsequent challenges. Supporting this notion, asphyxiated rat pups subjected to LPS (Lipopolysaccharide) exposure exhibit amplified IL-6 (Interleukin-6) production and persistent dendritic spine loss.

In preterm fetal sheep, moderate to severe hypoxia-ischemia results in profound neuronal loss in the hippocampus shortly after the insult, which is accompanied by transient microglial upregulation in the gray matter [19]. The fetal sheep model of moderate hypoxia-ischemia reveals spatiotemporal specificity in microglial responses: while microglia transiently increases in gray matter, peaking at 72 h post-insult, and white matter tracts exhibit delayed but sustained microgliosis (>14 days), correlating with myelination deficits [20]. Single-cell RNA sequencing identifies reactive subsets, including a pro-phagocytic population involved in synaptic stripping and an inflammatory cluster driving excitotoxic neuronal death [21].

The stress-induced microglial priming establishes a vulnerable neurodevelopmental window spanning late childhood through adolescence. During this period, secondary environmental insults, including viral infections, environmental toxins, and psychosocial stressors, may exacerbate neuroimmune activation through feedforward mechanisms. Emerging evidence [22,23] suggests that psychological stress drives neuroinflammatory programming via three principal pathways (Figure 1C), including (1) mitochondrial ROS (reactive oxygen species) overproduction: stress-induced oxidative phosphorylation (OXPHOS) uncoupling generates superoxide bursts, activating canonical NF-κB pathways through IKKβ-mediated phosphorylation of IκBα; (2) purinergic signaling dysregulation: extracellular ATP accumulation triggers P2X7 (purinergic receptor P2X, ligand gated ion channel 7) receptor activation, leading to NLRP3 inflammasome assembly via ASC (apoptosis-associated speck-like protein containing a CARD) speck formation and caspase-1 maturation; and (3) epigenetic reprogramming: DNMT3a-mediated hypermethylation at IGF1 promoter regions induces persistent transcriptional silencing, creating lifelong neurotrophic deficits through impaired BDNF-TrkB transactivation.

These molecular adaptations collectively establish a primed microglial state characterized by lowered activation thresholds and exaggerated inflammatory responses. This maladaptive priming represents a mechanistic nexus linking perinatal adversity to later-onset neuropsychiatric disorders, contributing to dysregulated synaptic pruning (excessive dendritic spine elimination) and disrupted excitatory/inhibitory balance within prefrontal-limbic circuits.

#### 2.1.2. Microglial miR-124 in Neurogenesis

Microglia further regulate neurogenesis through microRNA (miRNA)-mediated epigenetic modification. miR-124, a key miRNA highly expressed in the CNS, plays a crucial role in neuronal differentiation and adult neurogenesis [24] (Figure 1D). In microglia, miR-124 is highly expressed under resting conditions but is downregulated upon activation [25]. This miRNA maintains microglial quiescence by suppressing the C/EBP-α-PU.1 axis, a regulatory pathway essential for microglial activation. Its downregulation permits the pro-inflammatory transition [26]. Knockdown of miR-124 leads to microglial activation, likely due to the loss of C/EBP-α suppression, which otherwise inhibits PU.1-driven inflammatory responses [27]. Furthermore, microglia release IGF1, which promotes cortical neuron survival, facilitates NPC differentiation, and protects immature oligodendrocytes from apoptosis [25]. Additional microglial-derived trophic factors, including BDNF, bFGF (basic Fibroblast Growth Factor), EGF (Epidermal Growth Factor), HGF (Hepatocyte Growth Factor), NGF (Nerve Growth Factor), and PDGF (Platelet-derived Growth Factor), play multifaced roles in neuronal development and maintenance [28].

#### 2.1.3. Apoptosis Regulation of Microglia in Development and AFB1-Induced Stress

During development, nearly half of neurons and glial cells, including oligodendrocytes, undergo programmed cell death [29]. Microglia actively migrate to these regions, recognizing apoptotic cells and interacting with motor neurons via TNFα (Tumor Necrosis Factor-alpha) and superoxide production during the respiratory viral infection [29]. In vivo, TREM2-expressing microglia enhances the phagocytosis of debris from damaged or apoptotic neurons, thereby influencing microglial proliferation, survival, and immune function in the CNS [30].

AFB1 (Aflatoxin B1) induces apoptosis in microglial cells through oxidative stress, as observed in the spinal cords of mice and BV2 microglia cell cultures [31]. In murine spinal cord microglia, AFB1 exposure reduces cell viability, increases oxidative stress markers, and depletes antioxidant reserves in a dose-dependent manner [32].

The transcription factor IRF8 (Interferon Regulatory Factor 8) plays a crucial role in microglial development and homeostasis by regulating apoptosis-related genes. IRF8-deficient mice exhibit excessive proliferation of various hematopoietic cell types, including granulocytes, myelomonocytic cells, and lymphoid cells, underscoring IRF8’s role in controlling progenitor differentiation [33]. Notably, microglial progenitors in IRF8-deficient mice experience increased apoptosis, highlighting its essential function in maintaining microglial populations [34].

#### 2.1.4. Crosstalk Between NPC and Microglia Function During Neurogenesis

Emerging evidence suggests microglia exert bidirectional, context-dependent regulation of neurogenesis through dynamic interactions with NPCs (Neural Progenitor Cells). This regulatory axis operates via two principal mechanisms (Figure 1E): modulation of NPC-microglia crosstalk through secreted factors and epigenetic control of neurogenic niches [35,36]. It is important to note that the NPC-based therapeutic approaches discussed in this article are derived from studies conducted on animal models of disease, not from clinical applications in human patients.

In autoimmune demyelination, transplanted NPCs paradoxically exacerbate reactive microgliosis while simultaneously promoting the resolution of apoptosis. NPC-derived CCL2 (Chemokine (C-C motif) ligand 2) recruits CCR2^+^ (Chemokine (C-C motif) receptor 2) microglia to lesion sites, amplifying phagocytic clearance of myelin debris. Concurrently, NPC secretion of TGF-β1 induces microglial IL-10 production, facilitating the efferocytosis of apoptotic lymphocytes [37]. In contrast, in traumatic brain injury (TBI), NPC transplantation mitigates neurotoxic microgliosis. Grafted NPCs upregulate microglial IGF1 synthesis, promoting synaptic preservation via the PI3K-Akt pathway [38]. Additionally, NPC-derived lactate shifts microglial metabolism from pro-inflammatory glycolysis (M1) to OXPHOS-dominated anti-inflammatory state (M2). Injection of induced NPCs into TBI murine brains reduces the density of inflammatory microglia while increasing the number of IGF1-expressing microglia, indicating a neuroprotective effect [35,39].

Moreover, endogenous NPC density inversely correlates with reactive microglia density in the demyelinated corpus callosum. Under pathological conditions, NPC ablation leads to a reduction in microglial numbers at injury sites [35,40,41]. Activated microglia release IGF1 and BDNF, promoting NPC differentiation, enhancing oligodendrocyte survival, and stabilizing newborn neurons. This regulatory dichotomy may explain the observed inverse correlation between endogenous NPC abundance and reactive microgliosis in demyelinating lesions. In NPC ablation models, the depletion of progenitors leads to sustained microglial activation, ultimately impairing remyelination.

### 2.2. Synaptic Pruning in Neural Circuit Optimization

**Figure 2:**
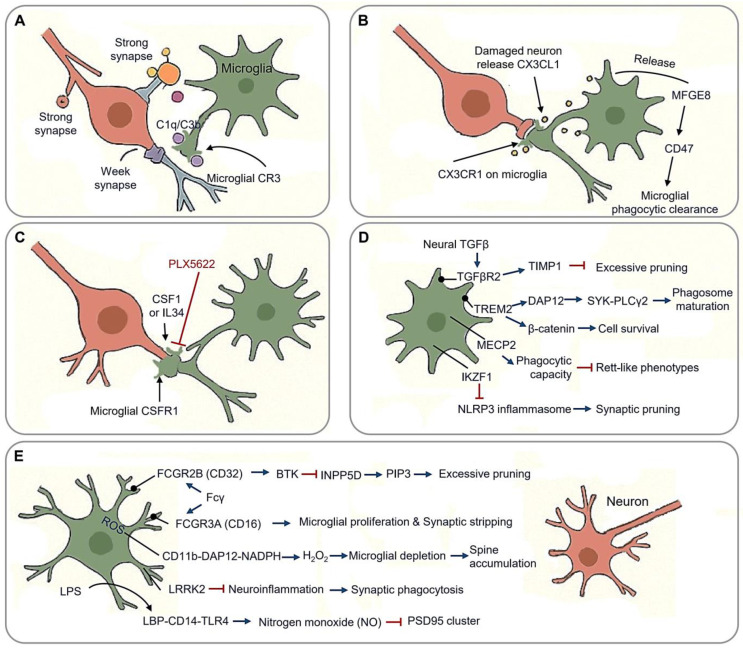
Synaptic pruning in neural circuit optimization. (**A**) Tag-and-eliminate strategy: microglia employ a “tag-and-eliminate” strategy in which complement cascade components (C1q/C3b/CR3) label weak synapses for phagocytic removal. (**B**) Neuronal CX3CL1 binds microglial CX3CR1, triggering dendritic spine engulfment via MFGE8 release. (**C**) Microglial phagocytic function is regulated by CSF1R homeostasis, a receptor system sustained by CSF1 and IL34; PLX5622 inhibits function of CSF1R. (**D**) Other molecular machinery of synaptic pruning. TREM2-DAP12 regulates microglial synaptic surveillance, and TREM2-β-catenin mediates survival; TGFβR2, MECP2 and IKZF1 involve microglial phagocytic function. (**E**) Synaptic stabilization and pathological engulfment through context-dependent molecular mechanisms. (Arrow: activation; red blunt arrow: inhibition; dot-headed arrow: receptor on the membrane).

Synaptic pruning, the elimination of redundant or dysfunctional neuronal connections, is a fundamental process through which microglia refine functional neural circuits. This activity-dependent mechanism occurs in three key phases: synaptic tagging via “eat-me” signals, phagocytic engulfment, and synaptic stabilization through trophic support. During critical developmental windows, microglia employ a “tag-and-eliminate” strategy in which complement cascade components (C1q/C3b) label weak synapses for phagocytic removal [42] (Figure 2A). This process peaks during adolescence, particularly in prefrontal regions, ensuring the retention of functionally relevant connections while eliminating approximately 40% of cortical synapses [43]. Ultrastructural analyses reveal that microglia preferentially engulf presynaptic terminals containing immature perforated synapses via phosphatidylserine receptor-mediated recognition [44].

#### 2.2.1. Molecular Machinery of Synaptic Pruning

Neuronal CX3CL1 binds microglial CX3CR1, triggering dendritic spine engulfment via MFGE8 (milk fat globule-EGF factor 8) release [45] (Figure 2B). CX3CR1 deficient mice exhibit increased immature dendritic spine densities, delayed critical period plasticity, and impaired long-term potentiation (LTP amplitude), underscoring the role of fractalkine signaling in synaptic pruning [43]. Damaged neurons release soluble CX3CL1, promoting microglial phagocytosis through the release of MFGE8, and facilitating apoptotic cell clearance through the integrin-associated CD47 signaling pathway [46].

The complement cascade plays a pivotal role in synaptic pruning via coordinated molecular mechanisms. C1q initiates the process by selectively tagging inactive synapses, enabling subsequent C3b opsonization. This tagging system enables precise targeting of synapses for elimination, with the CR3 receptor mediating downstream phagocytic activity through phagocytic cup formation. The essential nature of this pathway is evident in CR3-deficient mice, which retain approximately 60% more retinogeniculate synapses compared to wild-type controls [47]. Similarly, mice lacking C1q or C3 exhibit impaired synaptic elimination, resulting in excessive synaptic connectivity [47]. CR3 deficiency reduces microglial phagocytic activity, disrupting synaptic refinement [48]. This cascade mechanism ensures activity-dependent refinement of neural circuits through sequential molecular recognition and effector functions.

The microglial phagocytic function is critically regulated by CSF1R (Colony Stimulating Factor 1Receptor) homeostasis, a receptor system sustained by CSF1 and IL34 in the CNS [49,50,51] (Figure 2C). Regional variations in these ligands shape microglial identity and activation states across neural circuits [52,53,54]. Inhibiting CSF1R prevents cortical synapse loss by reversing microglial activation and reducing synaptic engulfment [55]. The pharmacological blockade of CSF1R with PLX5622 disrupts synaptic pruning, decreasing engulfment rates while preserving presynaptic terminals in the prefrontal cortex [56]. Notably, CSF1R inhibition prevents cortical synapse loss by suppressing microglial activation and phagocytic capacity, mirroring observations in human CSF1R loss-of-function mutations, which cause severe microglial depletion and white matter pathology [57]. However, developmental outcomes of CSF1R mutations differ between mice and humans, suggesting compensatory mechanisms or functional redundancy in non-microglial cell types during neural circuit refinement [58]. This highlights the context-dependent interplay between CSF1R signaling and synaptic remodeling across species.

Complementing this phagocytic machinery, neuronal IL33 directs activity-dependent extracellular matrix (ECM) remodeling via microglial MMP9 (Matrix Metallopeptidase 9) upregulation, and IL33 deficiency results in pathological perineuronal net accumulation and impaired fear memory extinction [59,60]. This neuron-to-microglia communication facilitates synaptic access through coordinated matrix degradation, illustrating how immune signaling actively shapes neural circuit plasticity.

The TREM2 (triggering receptor expressed on myeloid cells 2)-DAP12 (DNAX activation protein 12) signaling complex orchestrates microglial synaptic surveillance via phospholipid recognition (PS/PI) at tagged synapses, triggering DAP12 phosphorylation and recruitment SYK-PLCγ2 effectors to drive phagosome maturation (Figure 2D). This pathway’s functional significance is underscored by the Alzheimer’s-associated TREM2^R47H^ variant, which delays synaptic debris clearance and disrupts TrkB (Tropomyosin receptor kinase B) receptor recycling, thereby reducing BDNF responsiveness [61]. DAP12 deficiency severely impairs synaptic plasticity through TrkB downregulation while paradoxically maintaining developmental microglial density, despite causing population declines in adulthood [62,63,64]. Murine and human mutations both reveal conserved neuro-osteological defects, emphasizing DAP12’s dual roles in microglial and osteoclast regulation [63].

TREM2 also sustains microglial homeostasis through β-catenin-dependent survival pathways. Loss of TREM2 induces cell cycle arrest and apoptosis via β-catenin suppression [31,65,66]. Its activation enhances dendritic cell viability, while reduced TREM2 expression correlates with diminished survival in both primary and BV2 cultures [65,67]. Recent evidence highlights soluble TREM2 (sTREM2) as a key regulator of microglial inflammatory responses and survival, suggesting neuroprotective functions beyond phagocytosis [68]. These findings collectively outline a molecular continuum linking synaptic remodeling to microglial survival mechanisms.

TGFβ (Transforming Growth Factor β) signaling exerts critical trophic regulation over synaptic stability and microglial homeostasis (Figure 2D). Microglial TGFβR2 (Transforming Growth Factor Beta Receptor 2) maintains synaptic integrity through TIMP1 (Tissue Inhibitors of Metalloproteinase 1)-mediated suppression of excessive pruning and SMAD3 (SMAD family member 3)-dependent BDNF synthesis. Conditional knockout of Tgfbr2 in Emx1-Cre models results in severe neurostructural deficits, including spine density loss in layer 5 pyramidal neurons and a reduction in parvalbumin (PV) interneurons. Beyond synaptic maintenance, TGFβ signaling regulates microglial maturation and population dynamics [7,55,69]. Genetic ablation studies reveal stage-specific effects: Tgfb1 knockout reduces microglial precursor numbers and identity markers from embryonic day 14.5 without altering yolk sac progenitors [7], while embryonic Tgfbr2 deletion diminishes microglial populations, contrasting with preserved proliferation upon postnatal receptor loss [70]. These findings position TGFβ as a multifunctional regulator coordinating neurodevelopmental refinement with innate immune homeostasis.

Microglial MECP2 (methyl-CpG binding Protein 2) dysregulation contributes to neurodevelopmental pathology (Figure 2D), as evidenced by Cx3cr1-CreER-driven conditional *Mecp2* knockouts, which exhibit reduced synaptic phagocytic capacity, cortical hyperconnectivity, and Rett-like phenotypes. This mechanistic link between microglial *Mecp2* deficiency and impaired synaptic pruning underscores its role in Rett syndrome pathogenesis, where defective phagocytic activity exacerbates circuit dysfunction [71,72]. Similarly, CSF1R-related disorders highlight the non-redundant role of microglial in neurodevelopment, as homozygous CSF1R mutations induce severe brain malformations, including corpus callosum agenesis and white matter abnormalities [73,74,75]. These findings underscore microglial gene regulatory networks as crucial mediators of synaptic refinement and structural brain integrity.

The IKZF1 (IKAROS family zinc finger 1) transcriptional checkpoint critically regulates microglial homeostasis by restricting NLRP3 inflammasome hyperactivation (Figure 2D). *Ikzf1*-deficient models exhibit elevated caspase-1 activity, aberrant synaptic engulfment, and spatial memory deficits. Mechanistically, *Ikzf1* deficiency triggers a pathological cascade wherein microglia develop dystrophic morphology, acquire disease-associated molecular signatures, and accumulate intracellular synaptic markers, correlating with reduced spine density and impaired LTP [76]. This dysfunction, being characterized by unchecked neuroinflammation and impaired phagocytosis, is exacerbated under LPS challenge. Intriguingly, *Ikzf1* overexpression paradoxically induces a hyporeactive, less phagocytic microglial state, suggesting its role in balancing immune vigilance and synaptic surveillance [76]. Collectively, IKZF1 emerges as a critical regulator linking inflammasome control to synaptic pruning precision in cognitive circuit maintenance.

#### 2.2.2. Synaptic Stabilization vs. Neurodegenerative Transformation

Microglia maintain a delicate balance between synaptic preservation and pathological engulfment through context-dependent molecular mechanisms (Figure 2E).

Synaptic stabilization is primarily governed by Fcγ receptor regulation. Activation of FCGR2B (Fcγ receptor Ⅱb, CD32) suppresses excessive pruning via BTK (Bruton’s Tyrosine Kinase) inhibition and INPP5D (Inositol Polyphosphate-5-phosphatase D, SHIP1)-mediated PIP3 hydrolysis, while FCGR3A (Fcγ receptor Ⅲa,CD16) promotes microglial proliferation and exacerbates synaptic stripping in multiple sclerosis models [77]. Developmental priming further modulates this equilibrium, early-life n-3 PUFA (Polyunsaturated Fatty Acids) deficiency induces long-lasting hippocampal microglial dysfunction and elevated TREM2 shedding, impairing synaptic tagging capacity [78,79]. Regional specialization adds another layer of complexity. Facial nucleus microglia exhibit unique regenerative potential via CSF1R autocrine-driven clonal expansion and Wnt5a^+^ repair clusters identified through spatial transcriptomics, contrasting with limited evidence for broad spatial heterogeneity [13,80,81].

The transition to neurodegeneration involves ROS-mediated synaptotoxicity, wherein the CD11b-DAP12-NADPH oxidase axis generates synaptic H_2_O_2_, leading to Purkinje cell loss and caspase3^+^ dendritic spine accumulation upon microglial depletion [82]. LPS neurotoxicity operates through LBP-CD14-TLR4 complexes, triggering nitrogen monoxide (NO) bursts that collapse PSD95 clusters, alongside TNFα-induced AMPAR (Alpha-amino-3-hydroxy-5-methyl-4-isoxazole Propionic Acid Receptor) internalization (miniature excitatory postsynaptic currents (mEPSC) frequency reduction) [83]. Paradoxically, methamphetamine exposure induces microglial apoptosis while suppressing IL-1β maturation despite NF-κB priming [84]. LRRK2 (Leucine Rich Repeat Kinase 2) mutations further amplify neuroinflammation, enhancing synaptic phagocytosis and lysosomal dysfunction. In LPS-treated G2019S mice, microglial ablation attenuates weight loss but exacerbate home cage hyperactivity, highlighting the complex interplay between neuroinflammation and neurodegeneration [85,86]. Collectively, these mechanisms illustrate how stress-induced microglial reprogramming disrupts synaptic-neuroimmune equilibrium, priming circuits for degeneration in regions with high amyloid burden or innate immune activity [87].

## 3. Microglia in Neurological Diseases

Microglia help to clear damaged neurons and restrain extracellular protein aggregates in neurodegenerative diseases [88]. However, excessive activation of microglia enhances the proinflammation state and accelerates tissue damage [89,90,91]. Primary microglial dysfunction is implicated in monogenetic microangiopathies associated with severe CNS pathologies, positioning microglia as a promising therapeutic target for neuropsychiatric diseases, such as Alzheimer’s disease (AD), Parkinson’s disease (PD), Huntington’s disease (HD), and amyotrophic lateral sclerosis (ALS).

### 3.1. Alzheimer’s Disease: Phase-Dependent Microglial Dysregulation in Aβ-Tau Synergy

**Figure 3:**
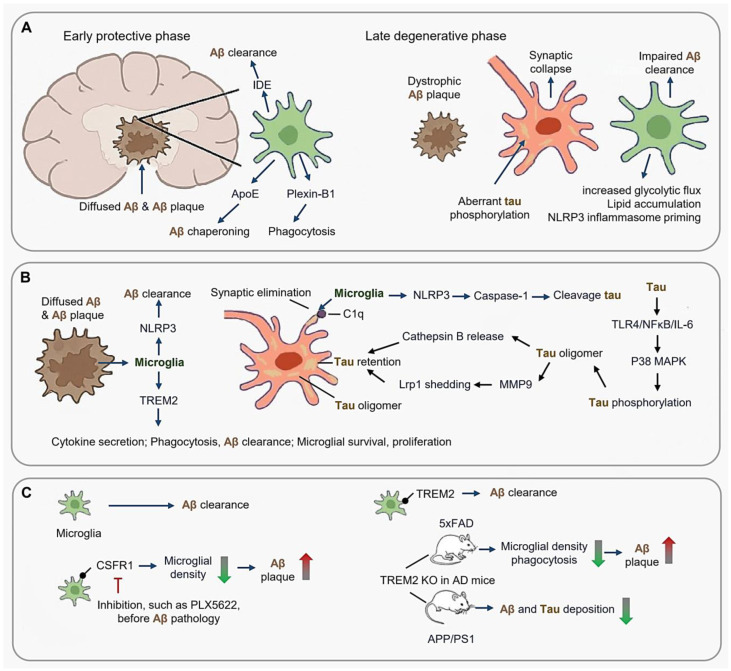
Phase-dependent microglial dysregulation in Alzheimer’s disease (AD). (**A**) Early protective phase and late degenerative phase. (**B**) Microglial response to Aβ plaques and tau-microglia crosstalk; (**C**) Therapeutic implications targeting microglia in AD. (Arrow: activation; Red blunt arrow: inhibition; Dot-headed arrow: receptor on the membrane; red thick arrow in panel (**C**): increase; green thick arrow in panel (**C**): decrease).

AD pathogenesis arises from dynamic interactions between amyloid-β (Aβ) plaques, tau pathology, and microglial dysfunction, with microglia exhibiting stage-specific roles in disease progression (Figure 3A). During the early protective phase, microglia mitigate Aβ toxicity through mechanisms such as IDE (Insulin-Degrading Enzyme)-mediated clearance, ApoE (Apolipoprotein E)-dependent chaperoning, and plexin-B1-driven phagocytosis of diffuse Aβ [92,93,94,95]. However, this neuroprotective capacity diminishes during plaque compaction, when Aβ42 fibrils trigger metabolic reprogramming, characterized by increased glycolytic flux, TREM2-dependent lipid accumulation, and NLRP3 inflammasome priming [96,97,98,99,100]. By the late degenerative phase, dystrophic microglia exhibit lysosomal alkalinization, impaired Aβ clearance, and aberrant tau phosphorylation, ultimately leading to synaptic collapse [101,102,103,104,105,106,107].

#### 3.1.1. Microglial Response to Aβ Plaques and Tau-Microglia Crosstalk

In both AD animal models and patients, microglia accumulate around the senile plaques [96,97]. Notably, rodent microglia display a greater proliferative capacity in vitro than human microglia [98,99], a distinction that manifests in vivo as well, with robust microglial proliferation observed in mouse models but not in human AD [29]. In AD mice, ablation of CXCR3 (Chemokine C-X-C motif receptor 3) ameliorates amyloidosis and cognitive decline [100] (Figure 3B).

TREM2 signaling is regarded to promote microglial proliferation, cytokine secretion, and phagocytosis, regulating microglial survival and metabolism [101,102,103]. Deletion of Trem2 in microglia reduces their phagocytic activity, survival, and proliferation while increasing pro-inflammatory cytokines [104]. Trem2 deficient AD mice exhibit enhanced Aβ deposition, reduced microglia around plaques, and decreased Aβ clearance [105]. Furthermore, TREM2 deficiency in microglia not only increases Aβ seeding and reduces microglial clustering but also lowers the expression of plaque-associated APOE [106,107].

Microglia also contribute to tau pathology through trans-synaptic propagation and inflammatory feedback loops. For instance, C1q marks hyperphosphorylated tau (p-Tau396) for complement-mediated synaptic elimination, while tau oligomers induce cathepsin B release and MMP9-driven LRP1 (Low-Density Lipoprotein Receptor Related Protein 1) shedding, accelerating parenchymal tau retention [11,61,108,109,110]. Concurrently, tau activates TLR4/NFκB/IL-6 signaling, stimulating neuronal p38 MAPK (Mitogen-activated protein kinase)-mediated tau phosphorylation [111]. Aβ-tau synergy is further reinforced by microglial NLRP3 activation, where caspase-1 cleaves tau at D421 and gasdermin D pores facilitate tau release tau, creating a self-perpetuating degenerative cycle [68,112,113,114,115,116,117].

#### 3.1.2. Spatiotemporal Evolution of Disease-Associated Microglia (DAM)

Single-nucleus RNA sequencing of human AD specimens reveals both parallels and discrepancies between mouse and human microglial transcriptional profiles [89]. Transcriptomic analyses have identified a distinct neurodegenerative signature, known as DAM (disease-associated microglia) or MGnD (neurodegenerative microglia), in plaque-associated microglia in both human and mouse models of Alzheimer’s disease, which is largely absent from non-plaque areas [11,61,108,109,110].

The identification of degenerating microglia has led to the microglial dysfunction hypothesis in AD; for instance, widespread microglial apoptosis observed in the AD brain, which suggests that the loss of microglial neuroprotection is central to the development of neurodegenerative diseases [113]. During the progression of AD, there is an initial reduction in microglial activation in subjects with mild cognitive impairment, followed by increased activation in AD patients; for instance, increased microglia numbers have been observed in 3-month AD mice [114].

#### 3.1.3. Therapeutic Implications

Microglia seem like a double-edged sword in AD because microglia are the main cells clearing Aβ plaque, but depletion of microglia such as CSF1R inhibition before Aβ pathology onset reduces Aβ plaque deposition in 5XFAD mice [115].

Therapeutic targeting of TREM2 remain controversial (Figure 3C). While TREM2 is essential for promoting the association between microglia and Aβ plaques, TREM2 deficiency or TREM2^R47H^ mutation results in reduced plaque-associated microglia and enlarged plaques [68,116]. TREM2-knockout experiments have produced conflicting outcomes in AD mice. In AD model 5×FAD mice, TREM2 knockout leads to suppressed proliferation, reduced numbers, and phagocytosis of microglia, causing the expansion of Aβ plaques [105]. Conversely, in another AD model APP/PS1 mice, TREM2 deficiency reduced amyloid and tau deposition [117].

### 3.2. Parkinson’s Disease: Microglial Orchestrators of α-Synucleinopathy

**Figure 4:**
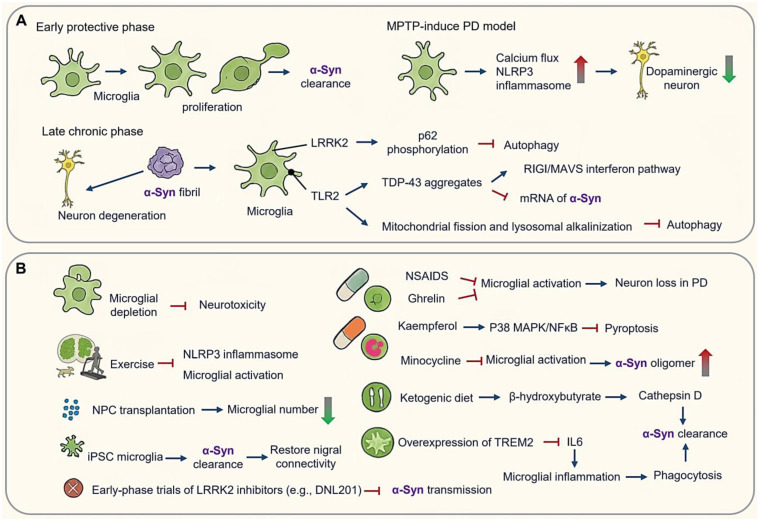
Microglial orchestrators of α-synucleinopathy in Parkinson’s disease (PD). (**A**) Phage-dependent microglial activation in PD pathogenesis. Early protective phase and late chronic phase. (**B**) Therapeutic implications and modulation targeting microglial checkpoints in PD. (Arrow: activation; red blunt arrow: inhibition; dot-headed arrow: receptor on the membrane; red thick arrow: increase; green thick arrow: decrease).

PD is driven by the dual onslaught of α-synuclein (α-Syn) proteopathy and the collapse of dopaminergic circuit, both of which are modulated by microglial functional states. Initially neuroprotective, microglia eventually transition into chronic propagators of α-Syn pathology through phase-locked molecular reprogramming, presenting both challenges and opportunities for therapeutic intervention (Figure 4).

#### 3.2.1. Phase-Dependent Microglial Activation in PD Pathogenesis

PD progression demonstrates distinct spatiotemporal microglial activation patterns. In early stages amenable to therapeutic intervention, microglia undergo pronounced proliferation accompanied by marked morphological changes. In acute MPTP (1-methyl-4-phenyl-1,2,3,6-tetrahydropyridine)-induced PD models, astrocytic conversion to MPP (mitochondrial-processing peptidase)-positive states facilitate dopaminergic neuron uptake, followed by microglial CX3CR1-mediated calcium flux and activation of NLRP3 inflammasome—events that precede dopaminergic neuronal loss [118,119] (Figure 4A). In postmortem samples of PD patients, an increase in reactive microglia has also been observed [120].

In chronic phases, α-Syn fibril propagation via LRRK2-dependent exosomes induces autophagy blockade through p62 hyperphosphorylation [121,122,123,124,125]. α-Syn impairs microglial autophagy and promotes neurodegeneration in a PD mouse model [125]. Pathogenic feedback loops emerge as α-Syn-TLR2 interactions drive mitochondrial fission and lysosomal alkalinization, while TDP-43 (TAR DNA binding protein) aggregates in PD microglia destabilize SNCA (Synuclein alpha) mRNA and activate RIG-I/MAVS interferon pathways [120,126,127]. Notably, TDP-43 proteins are not toxic to cultured motor neurons in the absence of microglia, underscoring that microglia mediate non-cell autonomous pro-inflammation [127].

#### 3.2.2. Therapeutic Modulation of Microglial Checkpoints

Microglial depletion abolishes neurotoxicity, while routine treadmill exercise attenuates microglial activation and reduces NLRP3/Iba1colocalization in MPTP-treated mice [123,124] (Figure 4B). Depletion models highlight a dual role of microglia: acute depletion mitigates neurotoxicity, whereas repopulation enhances neuroprotection through cytokine resetting, suggesting phase-specific therapeutic windows [122,123,124] (Figure 4B).

In an AD mouse model, NPC transplantation increases microglia numbers, while in mouse models of PD, spinal cord injury, or prenatal white matter injury, NPC transplantation decreases the microglial density [35,128,129] (Figure 4B).

Non-steroidal anti-inflammatory drugs (NSAIDs) reduce activated microglia by approximately 15% in PD models, and ghrelin prevents microglial activation, reducing pro-inflammatory molecules and inhibiting dopaminergic cell loss in a PD mouse model [130]. Kaempferol (KAE), a natural flavonoid, has shown neuroprotective effects in PD models by inhibiting microglial pyroptosis via the p38MAPK/NF-κB pathway [126] (Figure 4B).

Microglial depletion downregulated both anti-inflammatory and pro-inflammatory cytokines, and microglial repopulation resulted in neuroprotection, suggesting that microglial repopulation could be a therapeutic strategy for PD [122]. Clinical interventions have yielded mixed outcomes. While minocycline reduced microglial activation, it elevated cerebrospinal fluid levels of α-Syn oligomers without improving motor symptoms in Phase Ⅲ trials. Conversely, early-phase trials of LRRK2 inhibitors (e.g., DNL201) have shown promise in reducing α-Syn transmission and slowing dopamine transporter decline [93,131] (Figure 4B).

Preclinical advances highlight metabolic reprogramming strategies. For instance, ketogenic diets enhance α-Syn clearance via β-hydroxybutyrate-driven upregulation of cathepsin D, improving motor deficits. Stem cell approaches utilizing induced pluripotent stem cell (iPSC)-derived microglia engineered with α-Syn-degrading nanobodies achieve 90% uptake of pre-formed fibrils and restore nigral connectivity. Additionally, TREM2 overexpression boosts phagocytosis and suppresses IL-6 production in α-Syn models. These findings underscore the importance of temporally precise interventions—suppressing microglial inflammasome activation during acute phases while enhancing reparative functions in chronic degeneration [35,128,129,130] (Figure 4B).

Despite encouraging preclinical findings, clinical interventions such as minocycline have not altered the course of early PD. Nevertheless, targeting microglial activation remains a promising therapeutic strategy across neurodegenerative diseases, including PD and ALS, where modulating the balance between neuroprotective and pro-inflammatory microglial states may bring functional recovery [93,131].

### 3.3. Huntington’s Disease: Microglial Dysregulation in Mutant Huntingtin Proteostasis

**Figure 5:**
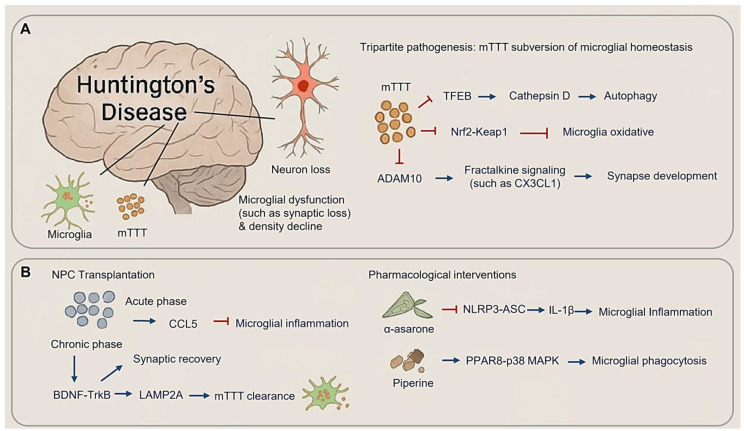
Microglial dysregulation in mutant Huntingtin proteostasis. (**A**) Microglial and neuronal change in Huntington’s disease (HD) (**Left panel**) and mTTT subversion of microglial homeostasis in HD. (**B**) Therapeutic rebalancing of microglial state in HD, including NPC transplantation (**Left panel**) and pharmacological interventions (**Right panel**). (Arrow: activation; red blunt arrow: inhibition).

HD is characterized by progressive neuron loss in the striatum and cortex, driven by the accumulation of mutant huntingtin (mHTT) aggregates. These aggregates disrupt microglial homeostasis and amplify neurotoxicity in a self-perpetuating cycle [35,132,133,134,135]. HD involves both microglial dysfunction and an age-related decline in microglia density [135]. Emerging evidence suggests that microglia function as both victims and contributors to mHTT proteostasis failure, where their compromised function exacerbates synaptic loss while simultaneously presenting potential therapeutic targets (Figure 5A).

#### 3.3.1. Tripartite Pathogenesis: mHTT Subversion of Microglial Homeostasis

HD pathogenic cascade arises from mHTT-induced dysregulation of microglial surveillance mechanisms, converging on three key mechanisms: autophagy-lysosomal dysfunction, oxidative stress, and impaired fractalkine signaling. mHTT aggregates disrupt proteostasis by sequestering TFEB (Transcription Factor EB), a master regulator of lysosomal biogenesis, thereby reducing cathepsin D activity and leading to autophagosome accumulation [136] (Figure 5A). Concurrently, the failure of Nrf2-Keap1 (Nuclear factor erythroid 2-related factor 2-Kelch-like ECH-associated protein 1) pathway exacerbates oxidative stress. This is evidenced by increased microgliosis and elevated oxidative DNA damage in Nrf2-deficient mice subjected to chronic cerebral hypoperfusion. Clinically, HD patients exhibit reduced levels of Nrf2 activators in cerebrospinal fluid, reinforcing the role of oxidative stress in disease progression [133,137] (Figure 5A). Additionally, mHTT impairs ADAM10 (A Disintegrin and Metalloprotease 10)-mediated fractalkine (CX3CL1) shedding. Levels of soluble CX3CL1 correlate with cortical synapse density, and cerebrospinal fluid concentrations predicting cognitive decline over three-year period in the ENCORE-HD trial cohort, positioning CX3CL1 as both a biomarker and a therapeutic target (Figure 5A).

#### 3.3.2. Therapeutic Rebalancing of Microglial States

Emerging therapeutic approaches aim to restore microglial function through mechanism-specific interventions. NPC transplantation demonstrates biphasic efficacy in HD models (Figure 5B): during the acute phase (0–4 weeks), CCL5 (Chemokine C-C motif Ligand 5)-mediated suppression of inflammation expands populations of repair-associated microglia populations; in the chronic-phase (8–12 weeks), synaptic density recovers via BDNF-TrkB signaling and enhanced mHTT clearance through LAMP2A (Lysosome-associated membrane protein 2)-driven chaperone-assisted autophagy [35,134]. Pharmacological interventions utilize plant-derived modulators, such as α-asarone, which inhibits NLRP3-ASC speck formation, reducing striatal IL-1β levels and slowing functional decline in Phase Ⅱ trials. Piperine activates PPARγ-p38 MAPK signaling, enhancing mHTT phagocytosis and restoring cortical gamma oscillations [138,139] (Figure 5B).

### 3.4. Amyotrophic Lateral Sclerosis: Spinal Microglial Dichotomy

**Figure 6:**
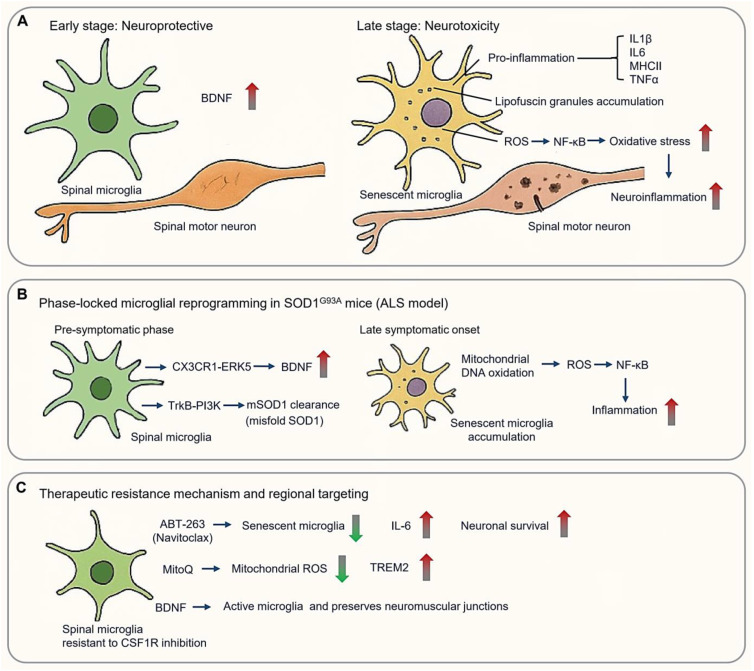
Spinal microglial dichotomy in amyotrophic lateral sclerosis (ALS). (**A**) Microglial biphasic response in ALS, from early neuroprotective (**Left panel**) to late-stage neurotoxicity (**Right panel**). (**B**) Phase-locked microglial reprogramming in SOD1 pathogenesis. (**C**) Therapeutic mechanism and regional targeting basing on spinal microglia resistant to CSF1R inhibition. (Arrow: activation; red blunt arrow: inhibition; red thick arrow: increase; green thick arrow: decrease).

ALS features a biphasic microglial response, transitioning from early neuroprotective responses to late-stage neurotoxicity (Figure 6A). In ALS early-stage, spinal cord microglia exhibit protective functions, including upregulation of brain-derived neurotrophic factor (BDNF). However, as the disease progressed, the microglia acquire a senescent phenotype characterized by the expression of pro-inflammation markers such as IL1β, IL6, MHCII, and TNFα, as well as the accumulation of lipofuscin granules. Mitochondrial DNA oxidation in aged microglia leads to ROS production, activating NF-κB signaling and exacerbating oxidative stress and neuroinflammation [29] (Figure 6A).

#### 3.4.1. Phase-Locked Microglial Reprogramming in SOD1 Pathogenesis

Spinal cord microglia demonstrate region-specific and temporal regulation distinct from cortical populations, shaping both disease progression and therapeutic resistance. Mutations in the *SOD1* (Superoxide Dismutase 1) gene are the first identified genetic causes of ALS [140]. Over 200 SOD1 mutations have been linked to fALS, typically inherited in an autosomal dominant manner. The SOD1^G93A^ transgenic mouse model, mimicking a pathogenic human mutation, remains a cornerstone of ALS research [141], closely found in approximately 20% of inherited ALS cases (Figure 6B). Intriguingly, spinal microglia in these mice maintained a neuroprotective phenotype throughout the disease development [142]. Selective depletion of proliferating microglia in the CNS of SOD1 mutant mice failed to affect disease progression, despite a >50% reduction in activated microglia in the lumbar spinal cord [142].

During pre-symptomatic phases (8–12 weeks), spinal microglia promote motor neuron survival through BDNF upregulation via CX3CR1–ERK5 (extracellular signal-regulated protein kinase 5) signaling and facilitate the lysosomal clearance of misfolded SOD1 via TrkB–PI3K pathways [143]. However, these protective functions collapse upon symptomatic onset (>16 weeks), when senescent microglia accumulate in the ventral horn. This is accompanied by mitochondrial DNA oxidation, and excessive ROS production, which fuels NF-κB-mediated neuroinflammation [29,142] (Figure 6B).

#### 3.4.2. Therapeutic Resistance Mechanisms and Regional Targeting

Spinal and cortical microglia display differential sensitivity to therapeutic modulation. Spinal microglia, for instance, demonstrate resistance to CSF1R inhibition, with only ~50% reduction in Iba1+ cells despite targeted treatment. This resistance is mediated by preserved CX3CR1^+^ neuroprotective subsets and compensatory activation of the CCL2-CCR2 axis [143]. Surviving microglia amplify IGF1 secretion, while astrocyte-derived GM-CSF sustains residual microglial niches [144]. Therapeutic strategies to overcome resistance include the use of ABT-263 (Navitoclax), which selectively eliminates senescent microglia, reducing CSF IL-6 and extending survival [145] (Figure 6C). MitoQ reduces mitochondrial ROS and restores TREM2 expression [146], while intrathecal delivery of TMEM119-driven BDNF selectively activates spinal microglia and preserves neuromuscular junctions more effectively than controls [147] (Figure 6C).

## 4. Therapeutic Interventions

### 4.1. Targeting Microglia for Therapy

**Figure 7:**
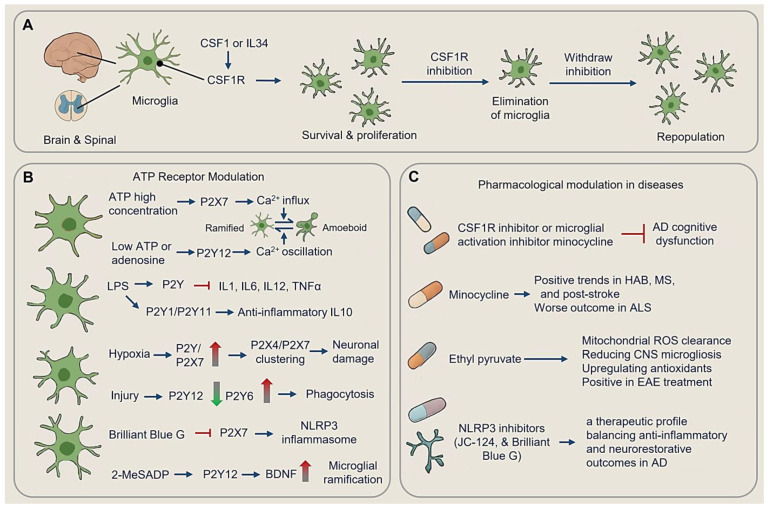
Therapeutic innervation targeting microglia. (**A**) Microglial CSF1R and microglial survival. (**B**) ATP receptor modulation. (**C**) Pharmacological modulation in different diseases. (Dot-headed arrow: receptor on the membrane; arrow: activation; red blunt arrow: inhibition; red thick arrow: increase; green thick arrow: decrease; LPS, lipopolysaccharide; ROS, reactive oxygen species; HAB, high-anxiety behavior; MS, multiple sclerosis; EAE, experimental autoimmune encephalomyelitis; CNS, central nervous system).

Understanding and manipulating microglial function holds promise for developing more effective disease-modifying treatments. As the primary resident immune cells and the first line of defense in the brain, microglia have garnered increasing attention due to their central roles in mediating neuroinflammation and immune responses [1,2,29] (Figure 7A).

#### 4.1.1. CSF1R and Microglial Survival

Although neurons show low CSF1R expression, microglia rely heavily on this receptor for proliferation and survival [106,148]. Throughout development and adulthood, CSF1 and IL34, as CSF1R agonists, sustain microglial populations and drive their proliferation [53,149].

Genetic and pharmacological approaches targeting CSF1R signaling have demonstrated the capacity to deplete microglia [49]. CSF1R inhibition leads to the near-total elimination of microglia from the adult CNS, without impairing behavior or cognition, and withdrawal of the inhibitor results in rapid repopulation of microglia [49]. Conversely, CSF1 overexpression induces aberrant microglial proliferation in mice [150].

Oral administration of PLX5622, a potent CSF1R inhibitor, achieves 78–90% microglial depletion in the cortex during early prion infection. However, microglial repopulation increases as the disease progressed, particularly around vacuolated regions where microglia cluster. In PLX5622-treated mice infected with RML scrapie, the extent of microglia depletion correlated with the accumulation of PrPSc (scrapie prion protein) and a reduction in survival time, underscoring the nuanced role of microglia in the disease progression [151].

#### 4.1.2. ATP Receptor Modulation

Microglial ATP receptor signaling exhibits concentration-dependent biphasic regulation of functional states, with morphological shifts serving as key indicators of polarization. At high concentrations, ATP activates ionotropic P2X7 receptors, triggering sustained Ca^2+^ influx that drives rapid transformation from ramified (homeostasis, resting phenotype) to amoeboid phenotypes. Amoeboid microglia are characterized by retracted processes, rounded cell bodies, increased migration speed, and priming pro-inflammatory cytokine production [152,153,154,155,156]. Conversely, low ATP/adenosine levels engage metabotropic P2Y12 (Purinergic Receptor P2Y, G Protein Coupled 12) receptors, inducing transient Ca^2+^ oscillations that gradually revert microglia to ramified states with branched processes and neuroprotective functions, including synaptic maintenance and debris clearance at surveillance speeds [156,157,158] (Figure 7B).

P2Y receptor subtypes differentially modulate inflammatory responses. In LPS-stimulated microglia, P2Y receptor activation suppresses IL1/IL6/IL12/TNFα release, while P2Y1/P2Y11 stimulation enhances the production of anti-inflammatory cytokine IL10 [157,158,159]. Hypoxia upregulates P2Y and P2X7 receptors, with glucose/oxygen deprivation promoting P2X4/P2X7 clustering, thereby exacerbating neuronal damage. In contrast, injury downregulates process-guiding P2Y12 while upregulates phagocytic P2Y6 receptors [160,161,162,163,164]. Pharmacological interventions exploit these dynamics. For instance, Brilliant Blue G (P2X7 antagonists) inhibit sNLRP3 inflammasomes and slows ALS progression in clinical trials, while 2-MeSADP (P2Y12 agonists) enhance microglial ramification and BDNF release in stroke models [160,163,164]. Disease-specific receptor remodeling further dictates therapeutic strategies: neuroinflammation hypersensitizes P2X7 and shifts adenosine A2A coupling to metabotropic Ca^2+^ signaling, while hypoxia induces P2Y1/P2Y13 upregulation, and traumatic injury rapidly activates P2Y6-mediated phagocytosis [160,161,162,163,164] (Figure 7B).

#### 4.1.3. Pharmacological Modulation

During experimental sepsis, microglial depletion with CSF1R inhibitor, such as PLX3397, or minocycline has been shown to alleviate cognitive dysfunction induced by Aβ oligomers, further underscoring the therapeutic potential of modulating microglial populations [165] (Figure 7C).

Minocycline treatment reduces microglial proliferation and activation [166]. Mincle Knockdown decreases microglia density [167]. Minocycline has shown positive trends in multiple sclerosis trials [168] but worsened disease outcomes in ALS patients, possibly due to its effects on the microbiota [169,170]. Minocycline treatment post-stroke improved motor function recovery without obviously affecting infarct volume or major features of microglial activation [171]. Early treatment altered the immune microenvironment, influencing the distribution of CD68-positive cells near the infarct rim [171] (Figure 7C).

Behavioral and pharmacological interventions dynamically reshape microglial activity across neuropsychiatric and neurodegenerative contexts. High-anxiety behavior (HAB) mice exhibit region-specific microglial remodeling, with increased microglial density in the dentate gyrus (DG) and elevated CX3CL1 levels in the medial prefrontal cortex (mPFC), correlating with anxiety phenotypes [172]. Minocycline alleviates these behavioral deficits by reducing DG microglial activation and normalizing exploratory behavior. Environmental enrichment further attenuates neuroinflammatory imbalances through running wheel-induced reductions in DG/mPFC microgliosis, restoration of IL-1β/IL-10 balances, and rescue of neurogenesis, demonstrating the plasticity of microglial responses to external stimuli [173] (Figure 7C).

Pharmacological strategies target redox and inflammasome pathways to modulate microglial states. Ethyl pyruvate, a mitochondrial ROS scavenger, reduces CNS microgliosis while upregulating Nrf2-dependent antioxidants, leading to clinical score reductions in experimental autoimmune encephalomyelitis (EAE) models [174] (Figure 7C). Resveratrol, known for its antioxidant properties, inhibits ATP- and LPS-activated NLRP3 inflammasome signaling, protecting microglia from oxidative stress and reducing pyroptotic cell death [175]. NLRP3 inhibitors, such as JC-124 and Brilliant Blue G, demonstrate multitarget efficacy through P2X7/NLRP3 dual inhibition, reducing Aβ-induced microgliosis in 5xFAD models while increasing synaptic density—a therapeutic profile balancing anti-inflammatory and neurorestorative outcomes [176] (Figure 7C).

### 4.2. Implications for Treatment Strategies

Microglial modulation strategies must account for dynamic functional states across disease progression. CSFs like GM-CSF (granulocyte-macrophage colony-stimulating factor) and M-CSF (macrophage colony-stimulating factor) act as potent mitogens, but their withdrawal induces microglial apoptosis via DNA fragmentation, highlighting the need for temporally controlled administration [177]. Therapeutic targeting of adenosine receptors reveals PKC (protein kinase C)-linked mechanisms: prolonged 2-chloro-adenosine exposure triggers DNA fragmentation independent of cAMP (cyclic AMP), inhibitable by H-7 and staurosporine but not dibutyryl-cyclic AMP, suggesting atypical receptor involvement [178]. These insights guide phase-specific interventions, GM-CSF neutralization with mavrilimumab reduces microglial proliferation during neuroinflammatory phases, whereas PEGylated M-CSF (Polyethylene Glycol-Modified Macrophage Colony-Stimulating Factor) enhances reparative phagocytosis through sustained release in late-stage neurodegeneration [177].

TREM2 exemplifies context-dependent therapeutic duality: early inhibition reduces neuroinflammation in pre-plaque AD, whereas late-stage agonists increase fibrillar Aβ compaction and microglial clustering to improve cognition [30,117]. Similarly, the IL6-TREM2 axis in demyelination models reveals therapeutic synergy—tocilizumab normalizes astrocytic IL6-driven TREM2 dysregulation, and when combined with TREM2 agonists, promotes remyelination and oligodendrocyte differentiation, as evidenced in phase Ib trials [179,180].

Precision targeting of adenosine receptors further underscores the importance of temporal optimization: acute-phase A2A agonism suppresses pro-inflammatory cytokines release, whereas chronic A1 antagonism enhances phagocytosis. Selective A2BR antagonists, such as PSB-603, inhibit PKC-dependent apoptosis, synergizing with neuroprotective agonists to maintain a balance between microglial survival and clearance [178].

## 5. Lifestyle Modifications

Emerging evidence underscores lifestyle factors as potent modulators of microglial function, offering non-pharmacological avenues to mitigate neuroinflammation and promote CNS resilience.

### 5.1. Polyunsaturated Fatty Acids (PUFAs)

**Figure 8:**
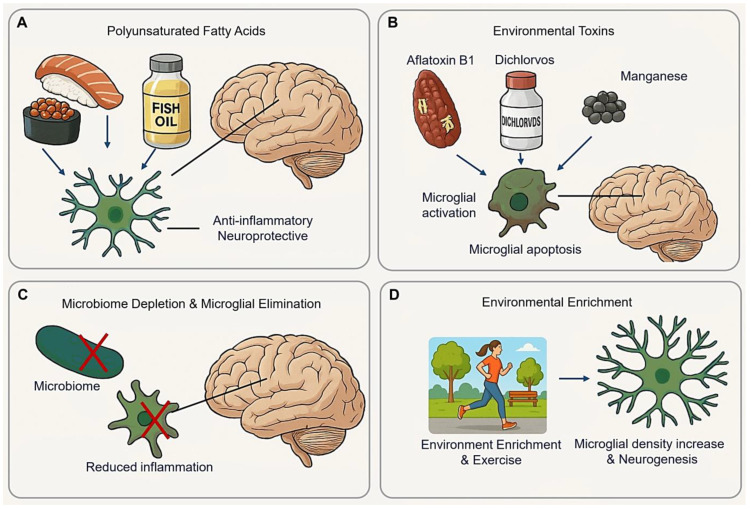
Lifestyle modification effect on microglia. (**A**) Polyunsaturated Fatty Acids exhibit anti-inflammatory and neuropeptide in the CNS. (**B**) Environmental toxins induce inflammatory and apoptosis of microglia. (**C**) Microbiome depletion changes microglial homeostatic state, reducing inflammation without severe side effects. (**D**) Environmental enrichment and exercise promote microglial proliferation and neurogenesis. (Arrow: activation; red cross mark: depletion or elimination).

Adequate consumption of Polyunsaturated Fatty Acids (PUFAs) is essential for CNS development and function. Long-chain n-3 PUFAs, such as docosahexaenoic acid (DHA) and eicosatetraenoic acid (EPA), exhibit anti-inflammatory and neuroprotective in CNS injury models, including traumatic brain injury (TBI) [181]. Higher brain DHA levels reduce neuroinflammation and improves recovery of post-TBI without altering microglia density or the M1/M2 phenotype [181] (Figure 8A).

### 5.2. Environmental Toxins

AFB1 (Aflatoxin B1) is widely present in moldy soil, plants, nuts, beans, dairy products, and cereals [182]. Acute oral administration of AFB1 reduces ascorbic acid and non-protein thiols in young rat brains, while chronic exposure increases catalase, MDA (malondialdehyde), and glutathione peroxidase activity [182]. Notably, prolonged AFB1 exposure induces astrocyte apoptosis, an effect mitigated by calcium chelators [183,184,185]. Additionally, AFB1 exposure significantly elevates free radical content in murine primary microglia [186], while naturally occurring AFB1 induces proapoptotic and inflammatory activation in human microglial cells [32,187] (Figure 8B).

Dichlorvos (2,2-dichlorovinyl dimethyl phosphate), a commonly used organophosphates pesticide in developing nations, induces neuronal cell death upon chronic exposure in rats. In primary microglial cultures, Dichlorvos triggers microglial activation and apoptosis, as supported by up-regulation of the pro-inflammatory molecules (nitric oxide, TNFα, and IL1β), the microglial activation marker CD11b, and apoptosis-related marker including Bax, Caspase-3, Cytochrome C release, and DNA fragmentation [188].

Manganese (Mn^2+^) exposure decreases microglial cell viability via ROS generation. Mn^2+^ induces necrosis in BV-2 cells through mechanisms involving parthanatos and lysosomal disruption [189]. The parthanatos pathway encompasses DNA damage, AIF (apoptosis-inducing factor) translocation, mitochondrial membrane permeabilization, and PARP1 (poly ADP-ribose polymerase 1)-dependent cell death. Additionally, Mg^2+^-induced lysosomal membrane permeabilization and cathepsin D release contribute microglial necrosis [189].

### 5.3. Microbiome Depletion and Microglial Elimination

The microbiome plays a significant role in regulating microglial function. For instance, Butyrivibrio fibrisolvens, a butyrate-producing bacterium, restore microbial homeostasis and prolong lifespan in SOD1 mice [190]. Under microbiome-depletion conditions, microglia may revert to a homeostatic state, as indicated by the presence of M0 homeostatic microglia in antibiotic-treated AD models [191]. Additionally, microbiome absence correlates with an increased microglial population during development [4,192] (Figure 8C).

Microglial elimination appears to be well-tolerated in adult organisms, with the safety of microglial ablation via CSF1R inhibitors confirmed in phase I and II clinical trials [193,194,195]. Furthermore, microglial ablation may relieve microglia-induced inflammation without inducing severe side effects [196].

### 5.4. Environmental Enrichment (EE)

EE has been shown to restore memory deficits and activate phagocytic microglia [197]. In 5×FAD mice, injected with 5×FAD brain homogenate and maintained in an EE, a significant increase in microglial density was observed in the dentate gyrus [197]. In wild-type mice, microglial numbers increased as expected [198], while in 5×FAD transgenic mice, the microglial response to EE depended on the presence or absence of PS1 mutations [199]. Furthermore, EE in adult rats enhances neurogenesis by increasing the number of neuroprotective microglia in the dentate gyrus [200,201] (Figure 8D).

## 6. Discussion

Studies in aged, non-demented humans have shown an increase in dystrophic microglia, characterized by abnormal morphological features indicative of senescence [202], suggesting that microglial dystrophy is a morphological reflection of senescence [113]. Microglial density and activation with age in regions such as the CA1–CA4 hippocampal subfields and the dentate gyrus [203]. Similar trends are observed in aged rhesus monkeys and chimpanzees, particularly in gray matter regions like the primary visual cortex [204,205,206,207]. However, findings in rodent models are inconsistent, with studies reporting region-specific increases, decreases, or no change in microglial activation with age [204,208].

Despite significant advances in understanding microglial biology, many questions remain regarding their precise roles in the onset and progression of neurodegenerative diseases. Current findings suggest that microglia exhibit disease-specific and stage-dependent phenotypes, but the mechanistic underpinnings of these dynamic states remain largely unexplored. Moreover, the causal relationship between microglial activation and neuronal dysfunction is still under debate in many disorders. Future research should prioritize the development of more physiologically relevant models, including human iPSC-derived microglia and brain organoids, to better recapitulate microglial behavior in human disease contexts. Additionally, single-cell multi-omics and spatial transcriptomics technologies will be essential for dissecting the functional heterogeneity of microglia and identifying novel therapeutic targets. Ultimately, a deeper understanding of microglial diversity and plasticity will be key to designing precise, disease-tailored interventions that modulate microglial activity to protect neural function rather than exacerbate degeneration.

In neurodegenerative conditions, microglia exhibit disease- and stage-specific alterations. For instance, AD is characterized by disease-associated microglia that respond differentially to amyloid-β and tau pathology, yet their phagocytic dysfunction accelerates synaptic loss. In Parkinson’s disease, microglia modulate α-synuclein clearance but sustain neuroinflammation via NLRP3 inflammasome activation, driving dopaminergic neuron loss. In HD, mHTT disrupts microglial proteostasis, impairing synaptic support and promoting striatal inflammation, while ALS reveals that microglial activation in motor cortices correlates with TDP-43 pathology and cognitive decline. Additionally, microglial dysfunction is increasingly recognized in, autism spectrum disorder, aging-related cognitive decline, and multiple sclerosis.

Therapeutic strategies targeting microglial states—such as CSF1R inhibitors, purinergic receptor antagonist, and microbiome interventions—highlight their potential as dynamic therapeutic targets. Lifestyle modifications, such as dietary adjustments and environmental factors, further underscore non-invasive avenues to optimize microglial function. These strategies illustrate that microglia are not merely passive bystanders but active regulators of CNS integrity and repair.

## 7. Conclusions

This review highlights the dualistic and dynamic nature of microglia across developmental and pathological contexts. While early sections emphasized the importance of appropriate microglial density during neurodevelopment, particularly in supporting neurogenesis and synaptic pruning, we also emphasized how deviations from this balance contribute to neurodevelopmental impairments and long-term vulnerability to disease.

In conclusion, therapeutic modulation of microglial activation and density offers promising avenues for managing neurodegenerative and neuroinflammatory diseases. Future research should continue to explore these dynamics to better harness microglia’s therapeutic potential, aiming to enhance CNS repair and protect against neurodegeneration mitigating harmful responses.
